# Linking Fold, Function and Phylogeny: A Comparative Genomics View on Protein (Domain) Evolution

**DOI:** 10.2174/138920208784139537

**Published:** 2008-04

**Authors:** Aartjan J.W te Velthuis, Christoph P Bagowski

**Affiliations:** 1 Institute of Biology, Department of Integrative Zoology, Leiden University, 2333 AL Leiden, The Netherlands; 2 Institute of Biology, Department of Molecular Virology, Leiden University Medical Centre, Albinusdreef 2, 2333 ZA Leiden, The Netherlands

**Keywords:** Domain, phylogeny, alignment, MUPP, PDZ, molecular evolution, protein folding, MPDZ, molecular modeling, multiple PDZ domain protein.

## Abstract

Domains are the building blocks of all globular proteins and present one of the most useful levels at which protein function can be understood. Through recombination and duplication of a limited set of domains, proteomes evolved and the collection of protein superfamilies in an organism formed. As such, the presence of a shared domain can be regarded as an indicator of similar function and evolutionary history, but it does not necessarily imply it since convergent evolution may give rise to similar gene functions as well as architectures.

Through the wealth of sequences and annotation data brought about by genomics, evolutionary links can be sought for *via *homology relationships and comparative genomics, structural modeling and phylogenetics. The goal hereby is not only to predict the function of newly discovered proteins, but also to spell out their pathway of evolution and, possibly, identify their most likely origin. This can ultimately help to understand protein function and functional relationships of protein families. Additionally, through comparison with transcriptional data, evolutionary data can be linked to gene (and genome) activity and thus allow for the identification of common principles behind fast evolving proteins and relatively stable ones.

In this review, we describe the basic principles of studying protein (domain) evolution and illustrate recent developments in molecular evolution and give valuable new insights in the field of comparative genomics. As an example, we include here molecular models of the multiple PDZ domain protein MUPP-1 and present a simple comparative genomic view on its structural course of evolution.

## COMPARATIVE STRUCTURAL AND FUNCTIONAL GENOMICS

The genome projects of the last decade have produced a staggering amount of sequence data, but most of the identified genes lack experimental determination of biological function or even in some instances identification. The advances in bioinformatics have allowed large-scale genome comparisons, and efforts are well under way to make similar use of comparative functional and structural genomic approaches. However, the wealth of comparative genomic data generated has yet to be followed by a comparable gain of structural and functional information.

The annotation of genes, the prediction of new genes and the allocation of regulatory elements to date largely relies on evolutionary relationships for which genome comparison is fundamental [1, 2]. In essence, comparative genomics is based on the assumption that the two (or more) analyzed genomes share a common ancestor and that the bases in the sequence of each organism are the result of evolution acting on the genome of this mutual ancestor.

In general, evolution forms and molds genomes through two processes, namely mutational forces that generate random changes (*i.e.*, point mutations or insertion-deletions [indels]) and selection pressures which can be positive, negative or neutral with regard to the presence of the mutation in the next generation [3, 4]. The combined effect of mutation and selection can subsequently be calculated and presented in a rate matrix, which denotes the probability of a mutation from one amino acid (or nucleotide) into another for a given period of time [5]. In turn, the rate matrix can be used to calculate alignments of two or more functional sequences. These functional sequences are, by definition, functions that are under evolutionary selection and are often a sequence of amino acids. However, they can, for example, also be transcription factor binding sites or RNA structures (*e.g.* microRNAs or viral RNA genomes). Commonly used rate matrices are BLOSUM and PAM [5, 6], which can readily be found implemented in BLAST and other well known sequence alignment programs [7-10].

As a result, a specific gene or protein of unknown function and biological importance can be compared to the sequence of a set of proteins with characterized functions. From these, the best matching group can be selected based on the number of domains and the nature of these domains. This information can be used to annotate the predicted gene or protein [2, 11-13].

Indeed, comparing genomes provides new insights into the biology of organisms whose hereditary material is under scrutiny. Some recent papers of comparisons between prokaryotes (*e.g.*, γ-proteobacteria) [14, 15], insects (*e.g.*, *A. gambiae* to *D. melanogaster*) [16, 17], mammals (*e.g.*, *M. musculus* to *H. sapiens*) [18, 19], but also more distant comparisons between yeast and human genomes [20] are good examples of this approach. Furthermore, these studies have shed light upon transcriptional regulation [21-25], horizontal gene transfer [14, 24, 26], conservation of proteome networks [20, 27, 28] and strain-specific adaptations [29]. The combined data in GenBank and other databases now covers sequences for over 200.000 species with at least 50 complete genomes, which makes numerous more genome comparisons feasible [30-32]. But comparative genomics, especially when combined with proteomics, protein folding and microarray data, offers far more than just that; it can be used to explicate the evolution of proteins and the structures that make up proteins: the domains. In this review we describe the approaches currently available to elucidate the evolutionary history of proteins and their domains. We also provide examples, based on the PDZ domains of the Multiple PDZ Domain Protein-1 (MUPP-1; MPDZ) [33] and the single PDZ domain protein Disheveled (Dsh) [34]. MUPP-1 is an important scaffolding protein, which could potentially play important roles in lipid raft assembly [35], in viral entry [36] and in cancer progression [37]. Dsh, with two different additional protein binding domains, a DIX and a DEP domain, plays a central role in development of invertebrates and vertebrates [38]. 

## SEQUENCE ALIGNMENT AND PHYLOGENY

Central biological features like metabolism, transcription and cell cycle progression are conserved from prokaryotes and single cell eukaryotes to humans [39, 40]. This conservation motivated and established the use of model organisms for studying conserved processes that are difficult or expensive to assess in higher organisms. Technological advances over the past two decades have led to the accumulation of genome-wide sequence data for many different species (see *e.g.*, http://www.ensembl.org), but in order to use these sequences they have to be compared to each other in either pair-wise alignments (*e.g.*, used in BLAST) or multiple sequence alignments, in which multiple sequences are compared simultaneously to each other (*e.g.*, employed in ClustalX, Phylip and Muscle (see Table **1**)).

Alignments can also be subdivided based on the terms *global* and *local*. When whole genomes are aligned, bases are lined up by inserting gaps in sequences to account for (hypothetical) insertions or deletions that have taken place since diversification from the common ancestor. Indeed, this can be performed from one end to the other, as global implies, but when working with small genomes of several thousand base pairs or with entire chromosomes of hundred million base pairs it will need processing power and will be time consuming. Therefore, it is mostly applied to relatively short gene or protein sequence data, although web-based alignments can also be browsed (*e.g.*, http://www.dcode.org). For the longer genomic nucleic acid sequences, a focus on regions of (local) high similarity is more feasible; the low sequence similarity regions are then ignored, which makes the procedure altogether much faster. 

Automated alignments commonly employ a scoring procedure to find the best alignment possible for the input sequences. This scoring takes into account the number of identical residues, the number of different residues, and the size and number of gaps present in the alignment. Each different residue and bigger or extra gap will result in a penalty. Additionally, different penalties are created for the differences between for example transversions and transitions; with the latter being more common and thus favored over transversions [41]. However, the optimized alignment may not be the true one, since parameters can vary from species to species [42]. It is therefore recommended to manually check alignments and improve them (see Table **1** for programs). Fig. (**1A**) shows an example alignment of a number of PDZ domains with different shadings representing the amount of conservation (100, 75 or 50%) at a particular position in the sequence.

Evolutionary distances can easily be estimated from small sequence alignments and can subsequently be used to create phylogenies, but also approximate divergence times, rates of evolution and ancestry sequences can be delineated from them. For phylogenetic analysis, multiple software packages are now available that often use one of these approaches: Maximum Likelihood [43, 44], Maximum Parsimony [7, 45], Neighbor Joining [7, 9] or Bayesian Estimation [46, 47] (see also Table **1**). To provide an example of such a phylogenetic tree, we used MrBayes to calculate, over 100,000 generations and a mixed rate matrix set, the best tree topology for the alignment given in Fig. (**1A**).

Since, the MUPP-1 protein of *Tetraodon nigroviridis* has 10 domains, *Xenopus tropicalis* 12 and *Homo sapiens* 13 one hypothesis could be that the last domain of the “ten domain structure” duplicated two to three times to make up for the extra 2 or 3 domains found in the higher vertebrates. If this holds true, the last three PDZ domains should cluster closely together in the phylogenetic tree. However, this appears to be not the case: *Tetraodon* *nigroviridis* PDZ 8 clusters with *Xenopus* *tropicalis* PDZ 9 and *Homo sapiens* PDZ 10, which suggests at least one domain duplication event in the middle of the protein. The separate clustering of *Xenopus* *tropicalis* PDZ 8 with *Homo sapiens* PDZ 8 points to an insertion event in their common ancestor, however. Of course, we can not exclude from this small analysis that the domain was already present in the very early vertebrates and only lost in *Tetraodon*. We will try to shine more light on this with a structural model of these events in Fig. (**2B**).

All phylogenetic information is extremely dependent on a proper alignment and not so much on the programs used to infer phylogeny [48]. Recently, software has been developed to combine the alignment procedure and phylogenetic analysis in one single program [47]. Current versions of this software can, however, only handle a limited set of sequences.

## PROTEIN DOMAIN CLASSIFICATION AND SUPERFAMILIES

By definition, a domain is a structural, functional, but also an evolutionary component of a protein. Domain duplication and reorganization play important roles in evolution. It has been estimated that at least 70% of the domains duplicated in prokaryotes. In eukaryotes this number is presumed to be even higher, ranging to up to 90% [49]. Not surprisingly, many proteins comprise of more than one domain [1, 50, 51].

Domains are essential and versatile evolutionary elements that have been used to create from a relatively limited set an enormous and diverse assembly of proteins. Many protein family resources (*e.g.*, Prosite and Pfam (see Table **1**)) present a hierarchical classification that is almost fully dependent on sequence similarity and motif identification. Close relatives, sharing for example >50% sequence identity and often also functional properties, are grouped into families and subfamilies (*e.g.* PRINTS (see Table **1**)). In turn, these families are grouped with other families into superfamilies [49, 52], with which they share for example ~25% sequence similarity. For a recent review on the function of these databases see reference [13]. 

## PROTEIN DOMAIN FOLDING

**Fig. (2):**
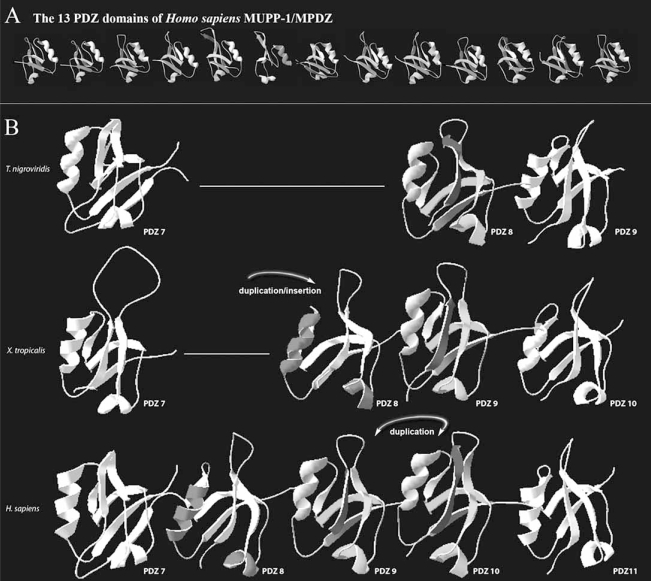
Structural modeling of MUPP-1 PDZ domains and hypothetical model for internal domain duplications. (A) Molecular modeling of the thirteen human PDZ domains of MUPP-1 with Swiss-Model Workspace and Swiss-PBD Viewer 3.7 [83]. (B) In figure **1B**, we compared the MUPP-1 PDZ domains of 4 different species. Of these four species, *Tetraodon nigroviridis* MUPP-1 consists of 10 PDZ domains, Xenopus tropicalis of 12 and *Homo sapiens* of 13 PDZ domains. Phylogenetic analyses implied that PDZ 8 of the *Tetraodon* MUPP-1 structure duplicated before at least twice to form the extra 2/3 PDZ domains present in the *Xenopus* and *Homo sapiens* structures. We therefore applied molecular modeling to these PDZ domains to visually support these findings. We modeled PDZ domains 7-9 of *Tetraodon nigroviridis*, domains 7-10 of *Xenopus tropicalis* and domains 7-11 of *Homo sapiens*. Indeed, PDZ 8 of *Tetraodon*, seems structurally related to 8 and 9 of *Xenopus* and 8-10 of *Homo sapiens*. However, within this group of six Xenopus PDZ 8 and Homo sapiens PDZ 8 appear to form a separate group. The most parsimonious explanation (and taking into account both the structural and phylogenetic data) therefore suggests one insertion event and one duplication event.

After sequence analysis, the question arises whether sequence divergence is correlated with structural divergence and ultimately functional divergence. In the 1970s technologies (NMR and X-ray crystallography) for determining the 3D structure of domains and proteins became established. It was found that protein structures are primarily composed of α-helical and β-strand secondary structures (see Fig. **2** for a PDZ domain model structure) and there usually is a clear way to achieve optimal packing of the hydrophobic residues in the core of the protein (or sometimes outside, in case of a transmembrane protein).

As the number of solved structures increased it quickly became evident that protein (domain) structures are much more conserved (~50%) than the protein (amino acid) sequence (~5%) [53]. For this reason, it is possible that protein structures and their models can be used to find close as well as very distant relatives. Indeed, sometimes it is difficult to recognize divergent relatives solely through sequence comparison and often for these cases, there are no features present indicative of mutual functional properties [54]. There are two possible explanations: both domains or proteins have evolved from two different ancestral proteins; or they are two extremely distant relatives that started out from the same evolutionary ancestor [50, 54]. To distinguish between these possibilities, it is important to look at the current understanding of domain evolution. It is believed that the small set of protein domains known to date, descended from an even smaller group of ancestral domains. Unlike the raw protein sequence, the core of the protein domain is largely stable as it must be functionally conserved (*i.e.*, selection is on function) and relies on inter-residue dependence. It is likely that protein evolution took place – or rather started – at the periphery of the relatively constant core. Indeed, it was shown that in pair-wise alignments, the amount of indels correlates with the evolutionary distance of proteins [4, 55, 56]. The structures most susceptible to point mutations, insertions or deletions are typically surface loops [57]. Unless mutations in these areas are neutralized, the number of changes will accumulate and eventually generate new polypeptide folds. Subsequently, positive selection will favor some of these newly arisen substructures when they become implemented in the biological process.

It should be clear from the above that the process of structural evolution is of a completely different order than that of sequence evolution, which is much faster. The tertiary sequence of a protein contains therefore much more phylogenetic signal and makes it far more likely to find linkages beyond the timeframe of standard sequence alignments [54]. Indeed, it may not be surprising that, like recognizing distinct sequence similarities, distinct folds and structures can be identified and classified as well. Examples are SCOP and CATH (see Table **1**), which are linked to the Protein Data Bank (PDB) that stores protein structural data. Moreover, structural information can be used to verify and support phylogenetic data. As an example we modeled the differently clustering PDZ domains of MUPP-1 (the phylogenetic analyses shown in Fig. (**1B**) implied one insertion and one duplication event to form the extra 2/3 PDZ domains present in the *Xenopus* [PDZs 8 and 9] and *Homo sapiens* structures [PDZs 8-10]). Indeed, PDZ 8 of *Tetraodon nigroviridis* seems structurally highly related to domain 9 of *Xenopus* and domain 9 and 10 of *Homo sapiens*. In other words, either of these structures appears more structurally related to the others in this small group than to any of the other (flanking) PDZ domains, which suggests duplication. The PDZs 8 of *Xenopus* and *Homo sapiens* form, however, a separate structural group as the phylogenetic analysis predicted. We therefore propose that the *Homo sapiens* PDZ 9 originates from a duplication event of the *Xenopus* PDZ 9 and that *Homo sapiens* PDZ 8 is a result of an insertion in the common ancestor of *Xenopus* and *Homo sapiens*. Our evolutionary model shown in Fig. (**2B**) can thus be used to confirm the phylogenetic tree shown in Fig. (**1B**).

Even though domains are recognized by prediction programs, like Pfam and SMART, the actual fold may be different due to intermolecular interactions. Proteins usually contain more than one domain (*i.e.*, multidomain proteins) and have evolved through a process of duplication and recombination of the limited set of protein domains available [51]. This principle not only brought together different enzymatic functions into single protein units (*e.g.*, a catalytic domain and an ATP binding domain resulting in a helicase or kinase), but also combined domains that could co-evolve into one larger superdomain. An example of the latter can be found in the MAGUK family of proteins in which the Src homology 3 (SH3) domain and the Guanylate Kinase (GUK) domain interact intramolecularly to form a superdomain involved in protein-protein interactions [58, 59]. Not surprisingly, the GUK domain in these proteins is often only partially active or lacks activity completely and it was recently found that this loss of GUK activity corresponds with a position further away from the origin in the phylogenetic tree of the MAGUK proteins [60, 61].

## GENES AND DOMAIN EVOLUTION BEYOND THE SEQUENCES

Important elements in a gene’s function are its spatial and temporal expression patterns. In recent years, microarray technology has made an extraordinary number of experiments possible that were aimed to map genome-wide expression levels under a variety of conditions [62-65]. For example, transcriptional comparisons have been made to look at for instance aging [66], pathogenicity [67] and non-coding RNAs [68]. Equivalent data is now, in addition to the sequence data, becoming available for dozens of different species and they provide a rich resource for comparative studies.

Unfortunately, the comparison of distantly related organisms can only be done under strictly defined expression conditions since gene expressions are not static. Indeed, by thoroughly controlling research conditions, comparisons between different (sub)species were made for conditions like embryogenesis, metamorphosis, sex-dependency and mutation rates [65, 69-72]. Other studies including diverse organisms such as yeasts, plants and primates, have revealed valuable information on promoter types and whether or not genes had previously undergone a duplication event [64, 65, 73, 74].

However, more evolutionary distant organisms may react differently to the same stimulus, which undermines comparison of gene expression data. To overcome this limitation, the association of co-expression data of genes and of expression signatures has been developed in addition to a direct comparison of individual gene expression changes [62]. Firstly, the co-expression between gene pairs is determined for each individual organism (within-species comparison) and this is then compared to the co-expression entities of other organisms. This approach focuses on the similarity and differences of the orthologous genes within their expression networks and this can be compared when species differences do not allow direct comparison at a specific condition. This system already has been applied for several species and it has revealed that both species-specific parts of the expression networks are combinations of conserved and newly evolved modules [62, 75, 76].

Another benefit of comparing co-expression of genes is that often functional entities can be discovered and, subsequently, new leads can be gained for functional interpretation. The approach can be combined with the search for common cis-regulatory elements at the promoter regions or applied to other similarity measures between genes, such as protein-protein interactions, phosphorylation networks or ligand-binding specificities [77-79].

## CONCLUDING REMARKS

Finding evolutionary relationships for genes, proteins or protein domains is mostly based on orthology and thus on best sequence matches. Identifying these and categorizing them depends largely on multiple sequence alignments and this will in most cases give good indications for function and fold. However, this approach usually discards apparent ambiguities that arise from species-specific duplications or losses and may therefore introduce extensive biases [80]. Biases may also derive from the method of alignment, the phylogenetic analysis and the sample size used [47, 48, 81]. Therefore, care should be taken to not regard orthology as a pure one-to-one relationship, but as a family of homologous relations [64] and to select for the appropriate method of analysis [48, 81].

Genome and proteome comparisons can be performed by looking at expression data and, preferably, co-expression patterns or protein-protein and phosphorylation interactions. In the end, it will be the ultimate challenge to combine all comparative data (sequence, structure, expression, interaction and function) into one biological network. Indeed, only through putting together data obtained from protein-protein interactions and co-expression networks, conserved functional cell cycle complexes shared among yeast, plants, worms and humans have been revealed [82]. Expectantly, with these approaches we will be able to clearly distinguish how different biological mechanisms integrate, mold and flow along the forces of evolution. This is certainly an exciting and stimulatory time for interdisciplinary genomic research.

## Figures and Tables

**Fig. (1):**
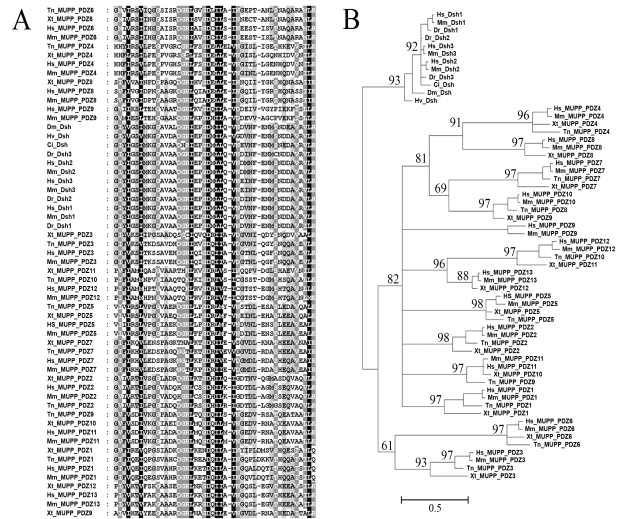
Example of sequence alignment and phylogeny. (A) Alignment of PDZ domains of the multiple (thirteen) PDZ domain protein MUPP-1 [33] and Disheveled (Dsh) [34] of several organisms. PDZ domains are modular interaction domains that recognize and bind to 4 C-terminal residues of the target domain, although other binding principles have also been shown. Black shading indicates 100% conservation, while the lighter grays indicates 75% or 50% conservation. Abbreviations used are Hs (*Homo sapiens*), Mm (*Mus musculus*), Xt (*Xenopus tropicalis*), Tn (*Tetraodon Nigroviridis*), Dr (*Danio rerio*), Ci (*Ciona intestinalis*) and Hv (*Hydra vulgaris*). (B) Evolutionary tree inferred by Bayesian Phylogeny (MrBayes) [46], rooted to the Dsh outgroup sequences. The tree shows clustering of most PDZ domains of MUPP-1 according to their sequence number in the protein. However, after PDZ 7 the numbers mix. Tn PDZ 8 clusters with Xt PDZ 9 and Hs PDZ 10, which suggests a domain duplication event. The clustering of Xt PDZ 8 with Hs PDZ 8 (near PDZ 4) and the separate clustering of Hs PDZ 9 suggest together one insertion (of PDZ 8) and one duplication event (of PDZ 9). To explain this most plausible relationship we have also presented this more visually in our structural model in Fig. (**2B**). It is important to note that MUPP-1 of *Tetraodon nigroviridis* contains 10, of *Xenopus tropicalis* 12 and of Homo sapiens 13 PDZ domains. Numbers indicated represent Bayesian posterior support values and all sequences used were obtained and analyzed as described previously [60].

**Table 1.** List of Resources and Databases Relevant to Comparative Genomics

Internet Resource	URL
**Sequence motif searches**	
Pfam	http://www.sanger.co.uk/Pfam/
PRINTS	http://www.biochem.ucl.ac.uk/bsm/dbbrowser/PRINTS/
Prosite	http://www.expasy.org/prosite/
SMART	http://smart.embl-heidelberg.de
Superfamily	http://supfam.mrc-lmb.cam.ac.uk/SUPERFAMILY/hmm.html
**Structural comparison**	
CATH	http://www.cathdb.info/latest/index.html
DaliLite	http://www.ebi.ac.uk/DaliLite/
SCOP	http://scop.mrc-lmb.cam.ac.uk/scop/
SSM	http://www.ebi.ac.uk/msd-srv/ssm/
**Alignment software**	
BLAST	http://www.ncbi.nlm.nih.gov/blast/Blast.cgi
ClustalW	http://www.ebi.ac.uk/Tools/clustalw2/
Dcode	http://www.dcode.org
MEGA	http://www.megasoftware.net/
Muscle	http://www.ebi.ac.uk/muscle/
T-coffee	http://www.tcoffee.org/
**Alignment optimization**	
GBlocks	http://molevol.ibmb.csic.es/Gblocks.html
GeneDoc	http://www.nrbsc.org/gfx/genedoc/index.html
PHYLIP	http://evolution.genetics.washington.edu/phylip.html
**Phylogenetic analysis software**	
MrBayes	http://mrbayes.csit.fsu.edu/
PhyML	http://atgc.lirmm.fr/phyml/
PHYLIP	http://evolution.genetics.washington.edu/phylip.html
Tree-Puzzle	http://www.tree-puzzle.de/
MEGA	http://www.megasoftware.net/
**Visualization**	
Pymol (structural)	http://pymol.sourceforge.net/
NJplot (phylogeny)	http://pbil.univ-lyon1.fr/software/njplot.html
MEGA	http://www.megasoftware.net/
Swiss-Model (structural)	http://swissmodel.expasy.org/
TreeView (phylogeny)	http://taxonomy.zoology.gla.ac.uk/rod/treeview.html
**Databases**	
Ensembl (Sanger project)	http://www.ensembl.org
PDB	http://www.rcsb.org/pdb/home/home.do
NCBI	http://www.ncbi.nlm.nih.gov/sites/gquery?itool=toolbar
Superfamily	http://supfam.mrc-lmb.cam.ac.uk/SUPERFAMILY/hmm.html
UniProt	http://www.expasy.uniprot.org/
